# Supplementary figures and images for: Increased yield of AP-3 by inactivation of asm25 in Actinosynnema pretiosum ssp. auranticum ATCC 31565
Source: PLoS One. 2022 Mar 22;17(3):e0265517. doi: 10.1371/journal.pone.0265517 (PMC8939807; doi:10.1371/journal.pone.0265517)

S1 Fig

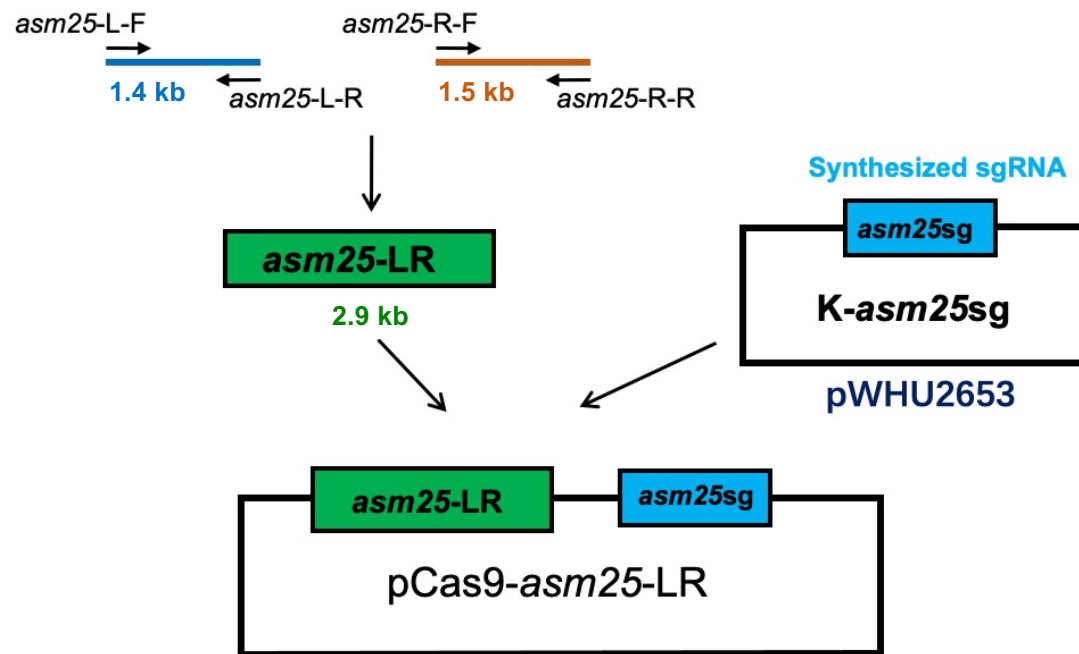

Supplement: S1 Fig — (PDF) [file pone.0265517.s001.pdf]

S2 Fig

A)

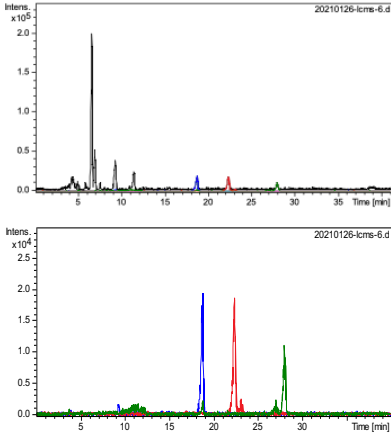

B)

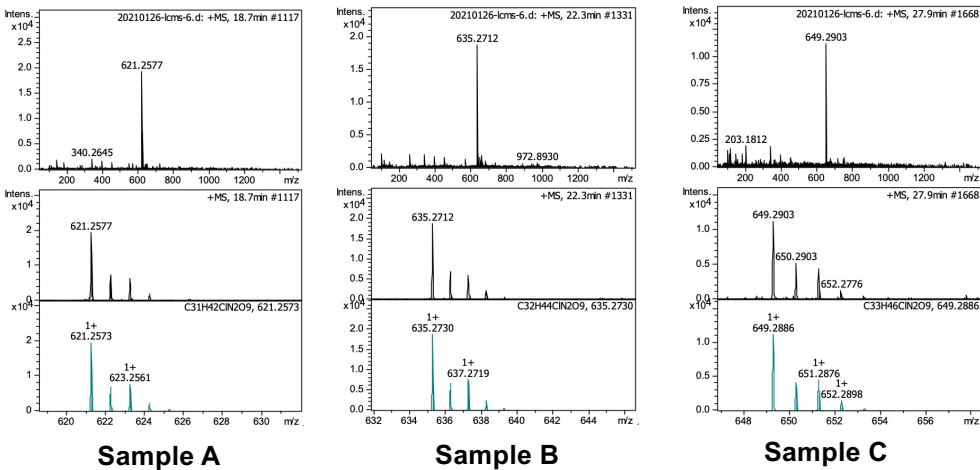

Supplement: S2 Fig — The products were analyzed and identified by Waters 600 series high performance liquid chromatograph (HPLC) and Bruker microtof-q II mass spectrometer (MS). The HPLC chromatogram and MS spectrum were well consistent with each other. Three samples, sample A, B and C, whose peak time ranged from 15 min to 30 min in MS spectrum, were further analyzed by hydrogenation analysis and compared with related database. Sample A, B and C were identified as AP-2, AP-3 and AP-4, respectively. (A) HPLC chromatograms. (B) MS spectrums. (PDF) [file pone.0265517.s002.pdf]

S3 Fig

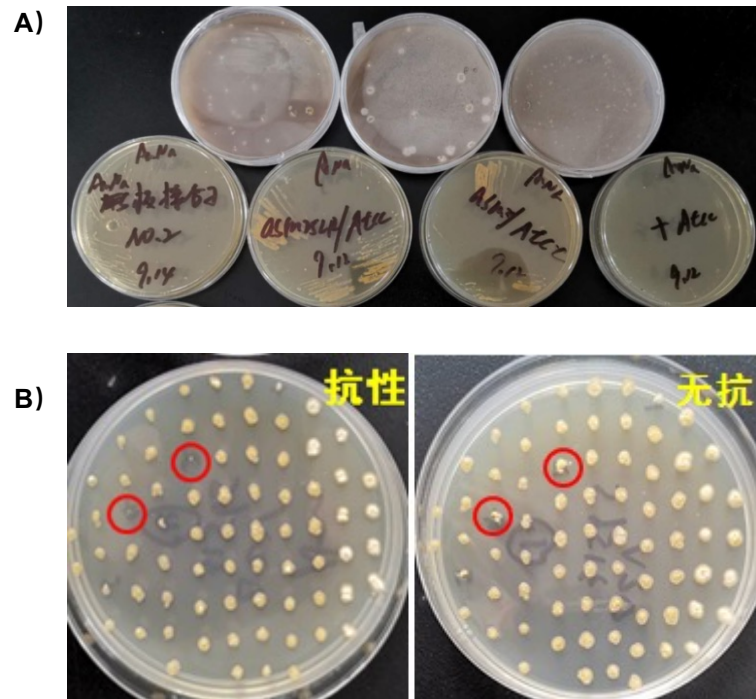

Supplement: S3 Fig — (A) Upper row: A. pretiosum ssp. auranticum ATCC 31565 cells mixed with plasmid-transformed E. coli ET12567 competent cells were plated on solid medium containing apramycin. Exconjugates were visible after 3–7 days. Lower row: Exconjugates single clones were sub-cultured for continuous three times on ISP2 solid medium without antibiotics to obtain knockout strain. (B) Exconjugants were plated on solid medium with or without apramycin to make the double-crossover clones visualized. Possible asm25 knockout strains were indicated by the red circles, which were further confirmed by PCR analysis. (PDF) [file pone.0265517.s003.pdf]

S4 Fig

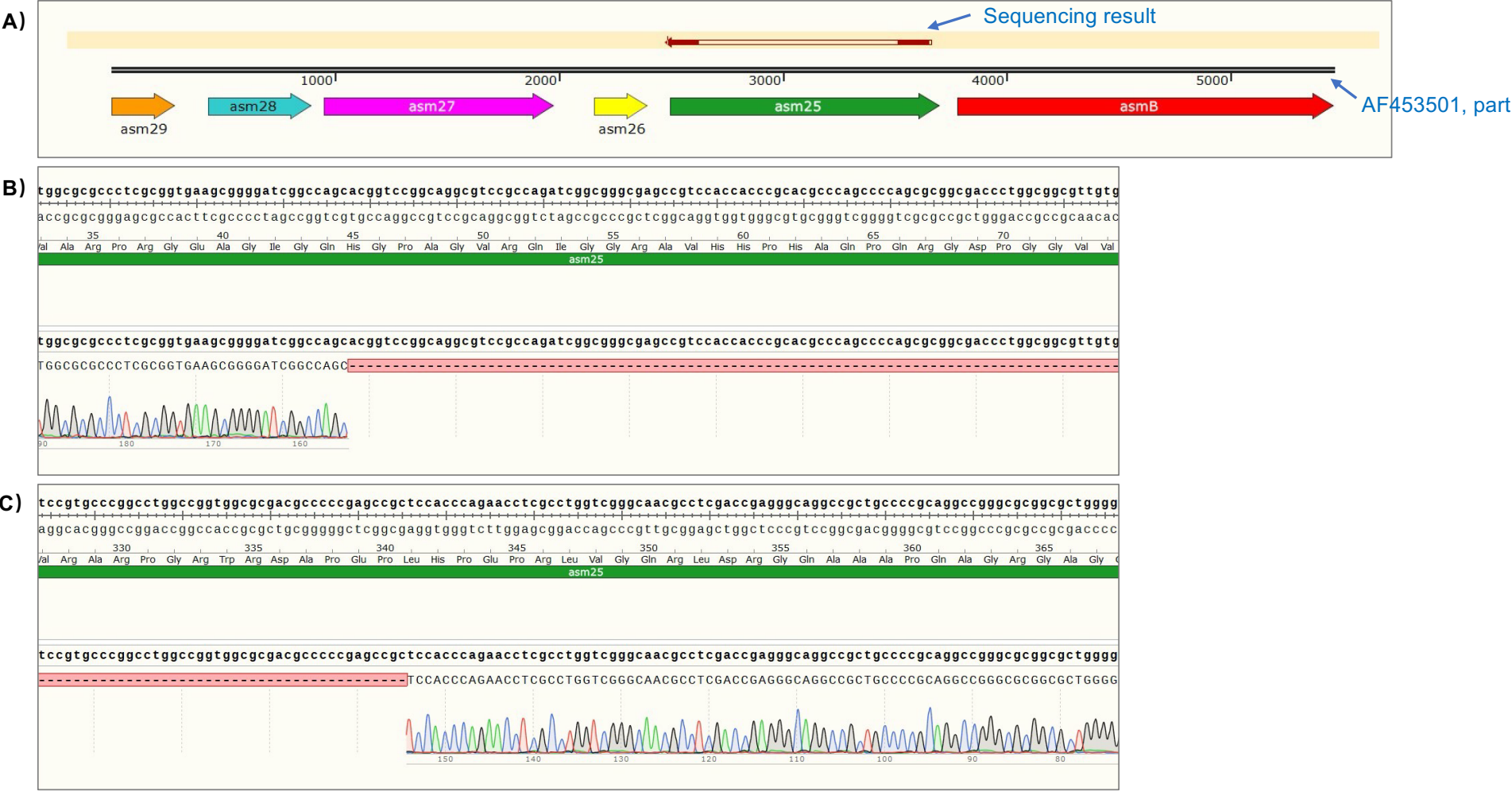

Supplement: S4 Fig — (A) Alignment between sequencing result (from asm25-seq-R) and AF453501 (Actinosynnema pretiosum subsp. auranticum maytansinoid antitumor agent ansamitocin biosynthetic gene cluster I, partial sequence, 82746 bp). A fragment of 885 bp was depleted from asm25 gene (885 bp of 1209 bp). (B, C) Alignment in detail by zooming in A. (PDF) [file pone.0265517.s004.pdf]

S5 Fig

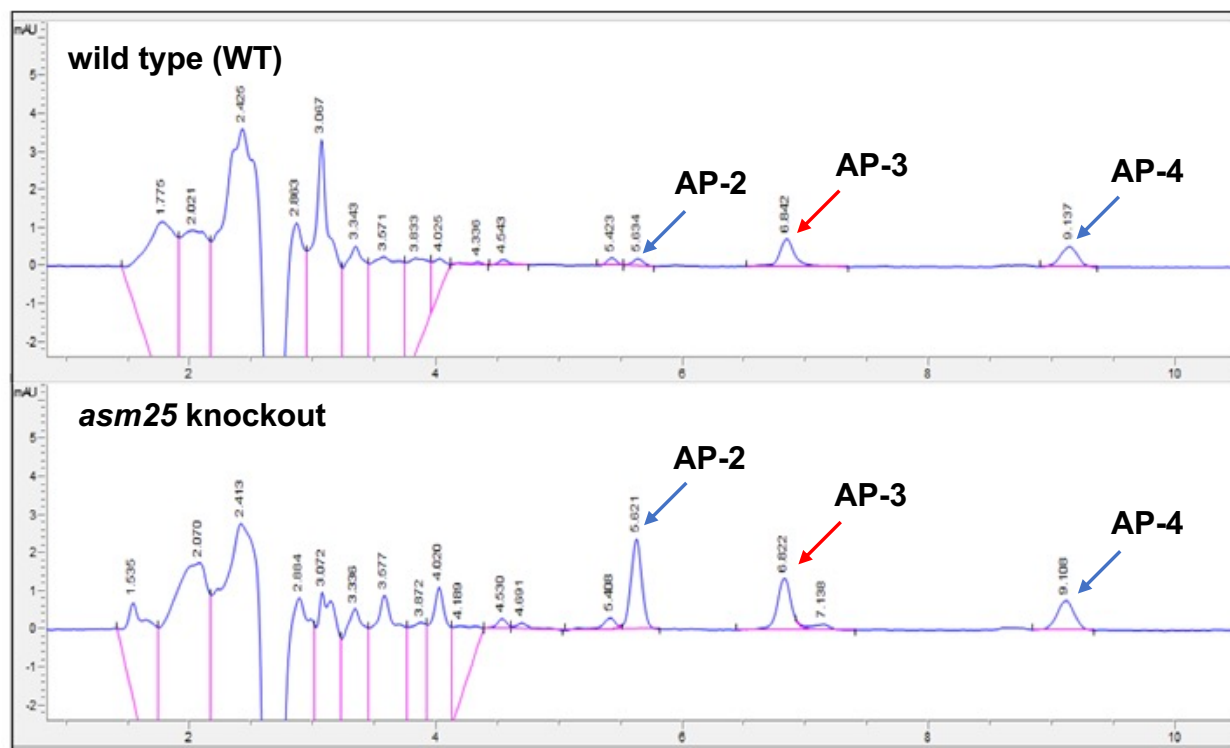

Supplement: S5 Fig — Red arrows showed AP-3, Blue arrows showed AP-2 and AP-4. (PDF) [file pone.0265517.s005.pdf]
